# A Combined Spectroscopic and In Silico Approach to Evaluate the Interaction of Human Frataxin with Mitochondrial Superoxide Dismutase

**DOI:** 10.3390/biomedicines9121763

**Published:** 2021-11-25

**Authors:** Davide Doni, Marta Meggiolaro, Javier Santos, Gérard Audran, Sylvain R. A. Marque, Paola Costantini, Marco Bortolus, Donatella Carbonera

**Affiliations:** 1 Department of Biology, University of Padova, Viale G. Colombo 3, 35131 Padova, Italy; davide.doni.2@phd.unipd.it (D.D.); marta.meggiolaro@studenti.unipd.it (M.M.); paola.costantini@unipd.it (P.C.); 2 Department of Chemical Sciences, University of Padova, Via F. Marzolo 1, 35131 Padova, Italy; donatella.carbonera@unipd.it; 3 Instituto de Biociencias, Biotecnología y Biología Traslacional (iB3), Departamento de Fisiología y Biología Molecular y Celular, Facultad de Ciencias Exactas y Naturales, Universidad de Buenos Aires, Intendente Güiraldes 2160, Ciudad Universitaria, Buenos Aires C1428EGA, Argentina; javiersantosw@gmail.com; 4 Departamento de Química Biológica, Facultad de Ciencias Exactas y Naturales, Universidad de Buenos Aires, Intendente Güiraldes 2160, Ciudad Universitaria, Buenos Aires C1428EGA, Argentina; 5 Aix Marseille Universitè, CNRS, ICR, UMR 7273, case 551, Ave Escadrille Normandie Niemen, CEDEX 20, 13397 Marseille, France; g.audran@univ-amu.fr (G.A.); sylvain.marque@univ-amu.fr (S.R.A.M.)

**Keywords:** frataxin, Friedreich’s ataxia, SDSL-EPR, fluorescence, protein-protein docking, molecular dynamics

## Abstract

Frataxin (FXN) is a highly conserved mitochondrial protein whose deficiency causes Friedreich’s ataxia, a neurodegenerative disease. The precise physiological function of FXN is still unclear; however, there is experimental evidence that the protein is involved in biosynthetic iron–sulfur cluster machinery, redox imbalance, and iron homeostasis. FXN is synthesized in the cytosol and imported into the mitochondria, where it is proteolytically cleaved to the mature form. Its involvement in the redox imbalance suggests that FXN could interact with mitochondrial superoxide dismutase (SOD2), a key enzyme in antioxidant cellular defense. In this work, we use site-directed spin labelling coupled to electron paramagnetic resonance spectroscopy (SDSL-EPR) and fluorescence quenching experiments to investigate the interaction between human FXN and SOD2 in vitro. Spectroscopic data are combined with rigid body protein–protein docking to assess the potential structure of the FXN-SOD2 complex, which leaves the metal binding region of FXN accessible to the solvent. We provide evidence that human FXN interacts with human SOD2 in vitro and that the complex is in fast exchange. This interaction could be relevant during the assembly of iron-sulfur (FeS) clusters and/or their incorporation in proteins when FeS clusters are potentially susceptible to attacks by reactive oxygen species.

## 1. Introduction

Frataxin (FXN) is a small, soluble protein that is highly conserved in most organisms, from bacteria to mammalian. The neurodegenerative disease Friedreich’s ataxia (FRDA; OMIM 229300) [1] results from low FXN expression, primarily caused by an abnormal GAA triplet repeat expansion in the first intron of the frataxin gene. In addition to low expression levels of FXN, several point mutations of FXN, including nonsense, missense, insertions, and deletions, have been associated with compound heterozygous FRDA patients [1,2,3,4]. The principal effect of FRDA is a large depletion of proteins containing iron–sulfur clusters (ISC) as cofactors, such as respiratory chain complexes (complex I-III) or other enzymes, such as aconitase. Protein depletion is also associated with a disruption of iron homeostasis with a marked increase in mitochondrial iron overload and increased levels of oxidative stress [5,6,7,8]. 

Nuclear-encoded human frataxin is synthesized in the cytoplasm as a precursor polypeptide of 210 amino acids; subsequently, it is imported inside mitochondrion where it undergoes a two-step maturation by the mitochondrial processing peptidase (MMP) [9,10,11]: MMP first cleaves a portion of the N-terminus, the mitochondrial import signal, producing the intermediate form (FXN 42–210), and in the second step, MMP produces the mature form (FXN 81–210), which is the most abundant form found both in normal individuals and FRDA patients [9]. The FXN C-terminus consists of a highly conserved block of about 100–120 amino acids, which is considered the most important part for protein function [12]. Residues 81–92 of the N-terminal portion are intrinsically unfolded [13] and are often partially truncated for in vitro studies [14] to obtain FXN 90–210. The structure of the folded portion of mature human FXN is relatively rare and consists of the sequence alpha-beta-alpha, where the two alpha helices are located side-by-side on the same side of the contiguous antiparallel beta sheet. FXN folding is remarkably stable, and the secondary structure has limited dynamics, as demonstrated by the high level of similarity between NMR [13] and X-ray crystallography [15] structures. The function of FXN is uncertain, and among the several proposed roles, FXN appears to act as a kinetic activator of the biosynthetic iron–sulpur cluster (ISC) machinery [16,17,18,19,20] and/or as a regulator of iron homeostasis [21] and ferroptosis [22,23], a recently identified iron-dependent form of cell death [24]. Furthermore, it has been suggested to be a protector against the oxidative damage commonly present in the pathological states of the mitochondrial respiratory chain [25,26,27,28]. 

Interestingly, in the context of the latter function, yeast FXN has been shown to interact with superoxide dismutases (SODs) [29]. SODs, a ubiquitous class of antioxidant enzymes, play a crucial role in the response to oxidative stress, catalyzing the dismutation of superoxide radicals into molecular oxygen and hydrogen peroxide. SODs are considered the first line of defense against reactive oxygen species (ROS), whose unbalanced concentration within the cell can dramatically contribute to the pathophysiological mechanisms of several diseases [30]. Up to now, three different SOD isoforms have been identified in mammals: two of them, SOD1 (or Cu/Zn-SOD) and SOD2 (or Mn-SOD), are cellularly localized, while the third isoform, SOD3 (or EC-SOD), is located in extracellular spaces [31]. SOD1 is mainly localized in the cytoplasm, although it is partially localized in the mitochondrial intermembrane space [30]. SOD2 is located exclusively in the mitochondria and exerts a pivotal role in the defense against superoxide radicals produced by electron transport chain complexes, the major source of ROS in cells. SOD2 is a homotetrameric enzyme containing one Mn^3+^ ion (in the resting state) per monomer as a cofactor.

In this work, building on previous in vitro work by Han et al. on the interactions between yeast FXN and bovine SOD1 and between yeast FXN and human SOD2 [29], we focus on the study of human FXN in its mature form (FXN 90–210) and human SOD2. Since human and yeast FXN show a good degree of homology, the interaction is likely to be conserved, and we use human proteins to confirm the interaction and gain a deeper molecular insight into the interface of the complex with a combined in vitro and in silico approach. We make use of the site-directed spin labelling technique (SDSL) coupled to electron paramagnetic resonance spectroscopy (EPR). This is a powerful tool for detecting changes in structure, dynamics, and oligomerization in proteins that was previously used to detect the effects of iron binding to FXN [32]. The EPR investigation is completed by endogenous tryptophan fluorescence experiments and Molecular Dynamics (MD) simulations. Finally, based on the experimental data, we perform a guided protein–protein docking between FXN and SOD2 to identify the interface region and suggest a possible structure for the FXN/SOD2 complex.

## 2. Materials and Methods

Materials Unless otherwise specified, chemicals were purchased from Merck and used without further purification. Ferric iron solutions were prepared from FeCl_3_·6H_2_O in HCl at pH 0.8. Human mitochondrial SOD (SOD2) was purchased as a His-tag recombinant protein from Technical Novusbio; the SOD2 buffer was Tris HCl buffer (20 mM, pH 8) containing 20% glycerol. The M-TETPO label was synthesized as previously described [32].

Heterologous expression and purification of human wild-type FXN and mutants A plasmid containing the coding sequence of human wild-type mature FXN, pET-9b/FXN(90–210), was previously obtained in our laboratory [33]. FXN mutants were obtained as previously described [32]. The two additional frataxin mutants used in this work, H177C and S202C, were obtained using the pET-9b/FXN(90–210) plasmid as a template and the couples of primers listed in the SI. Each sequence was checked by DNA sequencing (at Macrogen). *Escherichia coli* BL21 (DE3) cells were chemically transformed with the plasmids of interest, and positive clones were selected by antibiotic resistance. Expression of the wild-type and mutant proteins was induced by adding 1 mM isopropyl-β-thiogalactopyranoside (IPTG) in LB medium and incubating the bacteria cultures at 30 °C overnight under constant stirring. Cells were then harvested by centrifugation at 5000× *g* and 4 °C for 20 min, resuspended in lysis buffer (25 mM HEPES, pH 7.0) supplemented with protease inhibitors (1 μg/mL pepstatin A, 1 μg/mL leupeptin, 1 μg/mL antipain, 100 μM PMSF) and lysed by French press. The supernatant fractions were isolated from cell debris by centrifugation at 17,000× *g* and 4 °C for 15 min and incubated with 10 mM EDTA for 1 h at 4 °C under gentle agitation. Proteins were purified by combining anionic exchange and size exclusion chromatographies. The first chromatographic step was performed using a cationic DEAE (Diethylaminoethyl) Sepharose column using 25 mM HEPES (pH = 7.0) as the buffer of equilibration and 25 mM HEPES (pH = 7.0), 1 M KCl as the elution gradient buffer. The fractions corresponding to frataxin, as assessed by SDS PAGE, were collected, pooled together, concentrated by centrifugal filters (Amicon Ultra Centrifugal Filter, 3000 NMWL, from Merck Millipore, Burlington, MA, USA), and purified by size-exclusion chromatography (SEC). For FXN mutants containing cysteines, incubation with 1 mM of dithiothreitol (DTT) was performed for 1 h at 4 °C after cationic exchange. The second purification step was performed on a Superdex 200 GL 10–300 column (from GE Healthcare, Chicago, IL, USA) equilibrated in a buffer containing 25 mM HEPES (pH = 7.0) and 50 mM KCl. To estimate the molecular weights of the protein samples, the column was equilibrated in the same buffer and calibrated with the standards thyroglobulin (669 kDa), ferritin (440 kDa), β-amylase (200 kDa), bovine serum albumin (67 kDa), carbonic anhydrase (29 kDa), and cytochrome c (12 kDa). The eluted fractions containing frataxin proteins were finally pooled together, and the molar concentrations of the protein samples were determined spectroscopically using ε_280nm_ = 26,930 M^−1^cm^−1^ for all mutants (molar extinction coefficients were evaluated with the ExPASy ProtParam tool). Protein purity and integrity were finally assessed by SDS-PAGE and Coomassie blue staining (see Supplementary Materials, Figure S1).

Spin Labelling Protein samples were labelled with M-TETPO for the EPR experiments. Purified proteins (at a concentration of 50 μM) were incubated with DTT in a molar ratio of 1:100 at 4 °C for 30 min under slow agitation. Excess DTT was removed from the samples with a PD10 desalting column (GE Healthcare) using 25 mM HEPES (pH = 7.0) and 50 mM KCl as the elution buffer. Proteins were labelled with a tenfold molar excess of M-TETPO (dissolved in acetonitrile) and incubated at 4 °C for 2 h in the dark under slow agitation. Labelled protein samples were concentrated by centrifugal filters (Amicon Ultra Centrifugal Filter, 3000 NMWL, from Merck Millipore), and excess spin label was then removed by gel filtration using a Superdex 200 GL 10 300 column (from GE Healthcare) and 25 mM HEPES (pH = 7.0) and 50 mM KCl as the elution buffer. M-TETPO-labelled proteins were finally concentrated by centrifugal filters to a volume suitable for EPR spectroscopic analysis, and their concentrations were determined by UV-Vis spectroscopy, as previously described.

SDS-PAGE electrophoresis and Western Blot analysis Protein purity and integrity were assessed by SDS-PAGE and Coomassie blue staining prior to any spectroscopic experiment reported in this work. Samples of each step of FXN expression and purification and a small aliquot of recombinant SOD2 were solubilized in a Laemmli gel sample buffer containing 5% 2-mercaptoethanol and heated for 10 min at 95 °C on a heating block. Samples were then loaded into precast 4–20% polyacrylamide gel (GenScript^®^ ExpressPlusTM PAGE, Piscataway, NJ, USA). The run was done at 100 volts using Tris-MOPS-SDS Running Buffer Powder GenScript^®^ as the running buffer. At the end of the run, the gel was incubated with Coomassie Brilliant Blue colorant and subsequently destained using a solution of acetic acid 7.5%–methanol 10%. Correct separation and the molecular weights of the proteins were estimated using a marker of molecular weight loaded on the same gel.

For SOD2, an immunoblot assay was performed to assess the identity of the protein (see the Supplementary Materials, Figure S2). After the electrophoretic run, the gel was transferred to a nitrocellulose membrane (Life Science) through a semi-dry Trans-Blot^®^ TurboTM Transfer System (BioRad, Hercules, CA, USA). The membrane was then blocked with 10% milk in Tris-buffered Saline (TBS) for 1 h at room temperature and subsequently incubated at 4 °C overnight with the primary antibody (alpha-SOD2-HPA001814, Merck KGaA, Darmstadt, Germany) diluted 1:1000 in Tris-buffered Saline with 0.05% Tween20 (TBS-T). After incubation with the anti-rabbit IgG HRP-conjugate antibody (A0545, Merck KGaA, Darmstadt, Germany) diluted 1:20,000 at room temperature for 1 h, the protein was visualized using Immobilon^®^ Forte Western HRP Substrate (Millipore) with an Imager CHEMI Premium Detector (VWR). 

Fluorescence Fluorescence experiments were performed on a FLS 1000 UV/Vis/NIR photoluminescence spectrometer (Edinburgh Instruments) with a 450 W Xenon Arc lamp for excitation at 285 nm and a PMT-980 detector. The Peltier controlled holder allowed measurement at 288 K under stirring. The sample compartment was under constant nitrogen flow to avoid condensation on the windows and to maintain an anaerobic atmosphere. Experiments were conducted using a fluorescence cuvette (117104F-10-40 from Hellma, Müllheim, Germany) with a 10 × 4 mm optical path length and a gas tight screw cap with a silicon septum. The buffer was a mix of the SOD buffer (910 μL of 20 mM TRIS-HCl, pH = 8) and FXN buffer (90 μL of 25 mM HEPES, pH = 7.0, 50 mM KCl) to mimic the composition of the buffer in EPR experiments. The final concentrations of the proteins were as follows: FXN, 1.4 μM; SOD2, 1.9 μM. 

Electron Paramagnetic Resonance (EPR) spectroscopy EPR spectra were recorded on an ELEXSYS E580 spectrometer equipped with a SHQ cavity, both from Bruker BioSpin GmbH (Rheinstetten, Germany). The experiments were performed at room temperature, typically using the following parameters: microwave frequency 9.86 GHz, microwave power 19 mW (attenuation 9 dB), sweep width 150 mT, center field 351.4 mT, conversion time 164 ms, time constant 82 ms, and modulation amplitude 1.6 mT (1024 points, 25 averages per spectrum). Samples were prepared by thoroughly mixing 18 μL of SOD buffer or SOD2 protein stock solution (both in 20% *v*/*v* glycerol) with 2 μL of FXN stock solution to give a final FXN concentration of 10 μM; the resulting solution was put in a glass capillary (internal diameter 0.8 mm) and measured under nitrogen gas flow. The experiments with Fe^3+^ were performed on the same solution as above with the addition of 1 μL of Fe^3+^ stock solution. As previously verified [32], this volume of acidic iron solution does not change the pH of the solution.

Simulation of EPR spectra the simulation of the EPR spectra allowed quantitative information on the mobility of the spin label to be obtained. To perform the simulations, one must know or estimate the nitroxide g-tensor (**g**) and hyperfine tensor (**A**) and then adopt a model of spin-label motion based on the stochastic Liouville equation [34]. In this work we describe the spin-label mobility in terms of an isotropic diffusion tensor **D**. While this model is simplified, it fully allowed us to rationalize our results for all mutants in terms of the rotational correlation time τ_c_, which is derived from the diffusion tensor: τ_c_ = 1/6D_iso_. We used the MultiComponentEPR827.vi program designed by Christian Altenbach to perform the simulations. The program is written in LabVIEW (National Instruments) and can be freely downloaded [35].

Protein–Protein docking The molecular docking simulations were performed using four docking software packages that are freely available as webservers on the web. All of them allow the input of one or more residues to be considered for the interface: ClusPro [36], ZDOCK [37], GRAMM-X [38], and PatchDock [39,40]. The generation of the docking poses, the potential energy function that evaluates the energy of each docking pose, and the additional steps performed to rank the final docking poses all depend on the individual program (for details, we refer the reader to the relative references). ZDOCK, GRAMM-X, and ClusPro are based on a FFT approach to probe a fine grid for the generation of the docking poses. PatchDock adopts a different approach to accelerate the generation of the possible docking poses by matching the surfaces of two proteins based on their geometric complementarity. All programs perform additional steps to score the docking poses, each characterized by a different function. Therefore, to compare the results, the top solutions of each program were pooled together and re-ranked using a common scoring function. Among the different possibilities, we chose to adopt CONSrank, a freely available webserver that allows the solutions from all programs to be easily scored at the same time without only minimal formatting on the output files [41,42,43]. The re-ranking was based on the frequency of inter-residue contacts appearing in the solutions. Visualization, analysis, and plotting of the docking models were performed using UCSF Chimera software, developed by the Resource for Biocomputing, Visualization, and Informatics at the University of California, San Francisco with support from NIH P41-GM103311 [44]. 

Molecular Dynamics simulations Molecular Dynamics (MD) simulations and the analysis of production runs were carried out using the YASARA Structure [45] on the following hardware: Processor Intel CORE i7 10,700 10th generation; SOCKET 1200 2.9 GHZ (Max 4.8 GHZ) 16 M cores/threads 8/16, 2; Memory Kingston HX426C16FB3/8G HyperX 2666 Mhz; Disc SSD Kingston A400 240GB SATA 7 mm; Linux Ubuntu 20.04 LTS 64 bit. The coordinates corresponding to wild-type FXN (PDB ID: 1EKG) were solvated, and standard minimization protocols were applied to remove steric clashes. The simulation cell was prepared by maintaining a 20 Å water-filled space around the protein with a density of 0.997 g/mL. The system (cubic cell, periodic boundaries, and 8.0 Å cut-off for long-range coulomb electrostatics forces) was neutralized with 0.9% NaCl, and the temperature was maintained at 298 K with a pH of 7.4. After the initial steepest descent minimization, unrestrained replicas of 100 ns MD simulations using an ff14SB Amber force field [46] were carried out with 2.50 fs time steps. Snapshots were saved every 0.1 ns. The root-mean-square deviation (RMSD), root mean square fluctuation (RMSF), and secondary structure content were calculated.

## 3. Results

### 3.1. Choice of Labelling Sites and Molecular Dynamics of FXN

**Figure 1:**
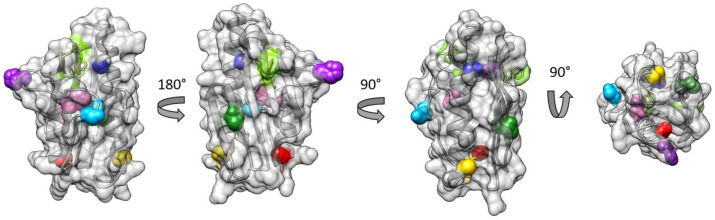
Structure of human FXN (pdb.id 1EKG) from different angles. The three native Trp residues are colored bright green and shown in stick representation. The spin labelling sites, A114 (yellow), T133 (green), H177 (purple), H183 (pink), A193 (red), and S202 (light blue), are shown in sphere representation. The third view from the left is the same as the one 0that appears below in the proposed models of the FXN/SODs complexes.

Mature FXN has a natively unfolded N-terminal region, followed by a region rich in Asp and Glu residues constituted by a long alpha helix and a loop, which constitute the main iron binding region that was characterized previously [47,48]. The beta-sheet region has been shown to be part of the interface in the ISC maturation complex, with W155 playing a key role [16]. We previously produced a library of FXN site-directed mutants across the protein where a native amino acid was mutated to cysteine and labeled with a spin probe to study the effect of Fe^2+/3+^ binding [32]. In this work, we used these mutants to investigate the possible interaction between FXN and SOD2 and produced two additional mutants to also map the loop region between the beta sheet and the short alpha helix (H177C) and the C-terminal portion of the protein (S202C). All positions where the spin labels were introduced are shown in sphere representation in Figure 1. 

**Figure 2:**
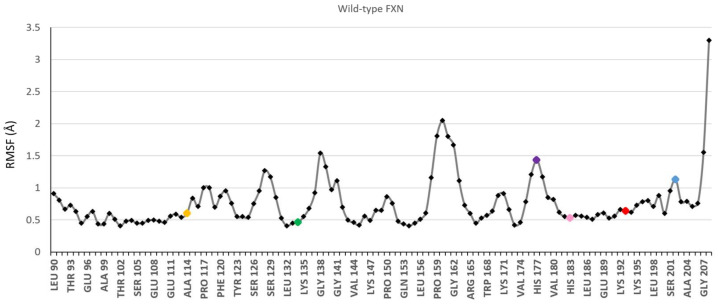
Root-mean-square fluctuations along the protein chain determined from the MD simulations on WT FXN (90-210). The spin labelling sites are shown as colored dots: A114 (yellow); T133 (green); H177 (purple); H183 (pink); A193 (red); S202 (light blue).

The local mobility of the residues, together with the tumbling of the protein, is reflected in the lineshapes of the EPR spectra of the spin probes. The internal motion of the wild type frataxin was studied by all-atom molecular dynamics (MD) simulations (nanosecond timescale) to investigate the mobility of the labeled positions (Figure 2 and Figure S6). The results are reported in Figure 2, showing the root-mean-square fluctuations (RMSF) along the protein chain; the mutated sites are shown in color using the same color code as used in Figure 1. The MD simulations clearly show that the H177 and S202 positions are more mobile than the rest of the labeled positions. Furthermore, H177 is part of a flexible stretch of the protein.

### 3.2. EPR Spectra of FXN Interacting with SOD2

We performed EPR experiments on FXN spin-labeled in different regions to try and pinpoint the interface formed by FXN and SOD2. The formation of an interface in the position where the nitroxide probe is located would result in a marked slowdown of side-chain motion, leading to a broader EPR spectrum. However, FXN is a small protein (MW = 13.64 kDa), and its tumbling motion in aqueous solutions at room temperature is rapid. The protein tumbling motion would mask any local contribution to the EPR spectral lineshape proper of the spin label; therefore, it was necessary to slow it down by carrying out the EPR experiments in viscous solution. We performed the experiments in 20% *v*/*v* glycerol, which slowed down the FXN tumbling enough to observe an EPR spectral shape influenced not only by the protein tumbling but also by the mobility of the spin probe sidechain and the backbone mobility. 

**Figure 3:**
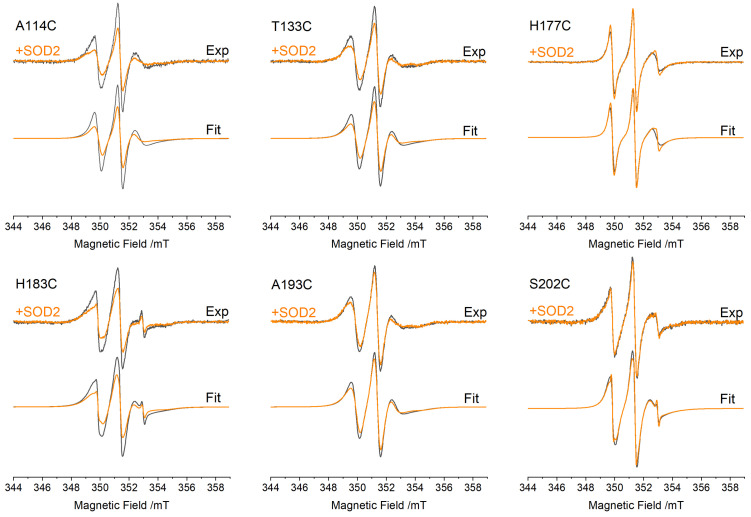
EPR spectra of FXN mutants (10 μM) in the absence (dark grey) and presence (orange) of SOD2 (40 μM) at a FXN:(SOD2)_4_ molar ratio of 1:1. For each mutant: top, experimental spectra; bottom, fitting. All spectra have been normalized to the same number of spins to compare the spectral shape in terms of the spin-probe mobility.

The EPR spectra of 10 μM FXN mutants in the absence or presence of 40 μM SOD2, giving a final FXN:(SOD2)_4_ molar ratio of 1:1, are reported in Figure 3. In the absence of SOD2, each mutant shows a characteristic spin label mobility, which confirms that the addition of glycerol slows the molecular tumbling of the protein enough that the spectrum reports both local mobility and general mobility. The mobility of the two new sites studied in this work (H177 and S202), as judged by the EPR spectra, faithfully reflects the high mobility of the native residues, as shown by the MD simulations presented in Figure 2. H177 is positioned in the loop between the beta sheet and the short helix and has the highest mobility among the studied residues, which is aided by the low level of crowding by nearby sidechains. Accordingly, it shows an EPR spectrum typical of very fast motion. S202 is in a relatively rigid part of the C-terminal region (residues 196–210), but it has greater mobility relative to neighboring residues, and its EPR spectrum indicates faster mobility than that of all other positions other than H177. 

Following the addition of an equimolar quantity of tetrameric SOD2, all positions were affected similarly, except for positions H177 and S202, for which the effect was almost null. Similar but slightly less pronounced effects were obtained with a FXN:(SOD2)_4_ ratio of 1:0.5, closer to that used in the fluorescence experiments reported below (see the Supplementary Materials, Figure S3). The spectral changes clearly indicate an interaction between FXN and SOD2.

To better quantify the effects of SOD2 on FXN, we fitted the EPR spectra by obtaining the rotational correlation time, τ_C_ (the fittings are reported in Figure 3 as grey and orange lines). The slow motion of the EPR probe was assumed to be isotropic. This simple model captures the spectral variations induced by SODs quite well and makes it easy to discuss the spectral variations in terms of the changes in the rotational correlation times (Δτ_C_), reported in Table 1 as the difference between τ_C_ in the presence of SOD (τ_C_^+SOD^) and the one in its absence (τ_C_^−SOD^). 

A homogeneous reduction in dynamic Δτ_C_ was observed for four sites (A114, T133, H183, A193) spread over a large region of the protein. The observed change likely reflects the slowdown in protein tumbling following the formation of the complex with bulky SOD2. In particular, H183, being an internal residue with limited sidechain and backbone dynamics, has the lowest mobility (highest τ_C_) of all sites and almost purely reflects the protein tumbling. Given that the changes in protein tumbling are the dominant effect on spin-label mobility, we cannot exclude the idea that one or more of the exposed sites (A114, T133, A193) are located at the interface between the two proteins. On the other hand, Δτ_C_ indicates that the mobility of H177, and S202 remains almost unchanged upon the addition of SOD2. Their mobility is always dominated by the fast backbone and sidechain motions (lowest τ_C_), making them unsensitive to protein tumbling changes. This excludes the idea that these latter residues partake in the protein–protein interface: if they were at the interface, their sidechain motion would be strongly reduced, bringing down their τ_C_ to that characteristic of protein tumbling (that of the internal site H183). 

Another important aspect that emerged from the simulation of the EPR spectra was that only one motional component was present in all spectra, even after the addition of SOD2, suggesting that the interaction between the proteins is in the fast-exchange regime.

Previously, we showed that the addition of excess iron slows FXN motion by inducing its aggregation at all positions [32]. Here, we tested the effects of Fe^3+^ addition in the presence of SOD2 by performing EPR experiments at a ratio of FXN:(SOD2)_4_:Fe^3+^ 1:0.5:20. The spectra, reported in the Supplementary Materials in Figures S4 and S5, clearly show that the presence of SOD2 has no effect on FXN aggregation at this molar ratio.

### 3.3. Fluorescence Spectra of FXN Interacting with SOD2

**Figure 4:**
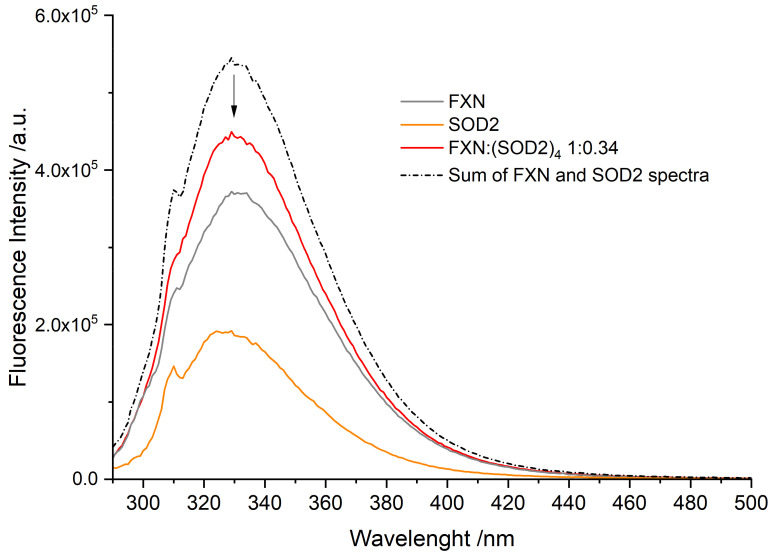
Tryptophan fluorescence spectra of WT FXN (1.4 μM) and SOD2 (1.9 μM). Temperature 288 K, λ_exc_ = 280 nm. FXN alone, grey; SOD2 alone, orange; SOD2 and FXN (molar ratio FXN:(SOD2)_4_ 1:0.34), red; sum of the individual fluorescence spectra of SOD2 and FXN, red. The peak at 310 nm is the Raman peak of the buffer.

The interaction between FXN and SOD2 was tested using the fluorescence emission of the tryptophan residues and comparing the fluorescence of the individual proteins with the fluorescence of the mixed solution. The results are reported in Figure 4. We must note that the spectra show the Raman scattering peak of the buffer at 310 nm (corresponding to a Raman shift $\bar{\nu \;}$ = 3456 cm^−1^).

Human FXN has three native Trp residues (W155, W168, W173); the Trp residues are shown in green in stick representation in Figure 1. While the W155 residue is in the beta sheet region and exposed to the solution, W168 is partially exposed and W173 is completely buried. It should be noted that all three Trp residues are spatially close together and, therefore, interact with each other via energy transfer, affecting the overall fluorescence in a complex way. W155, being the only fully exposed Trp residue, is likely the most sensitive to the presence of quenchers in solution. We must note here that, in our previous study, we verified that fluorescence experiments based on Trp quenching may be not fully reliable when the quencher binds far away from the Trp region [32]. For Frataxin, the fluorescence spectrum, therefore, results in a peak at 332 nm (grey line in Figure 4).

Human SOD2 has six Trp residues per protein chain (W78, W123, W125, W161, W181, and W186), most of them buried in the protein interior and in proximity to each other, either in the same chain or in neighboring chains in the tetramer, with only W181 and W186 exposed to the solution. SOD2 fluorescence is largely self-quenched, and the limited solvent exposition leads to it being blue-shifted relative to FXN with a peak at 328 nm (orange line in Figure 4).

The fluorescence spectrum of the mixed FXN:(SOD2)_4_ solution at a molar ratio of 1:0.34 (red line in Figure 4) shows marked quenching relative to the sum of the experimental spectra of the individual proteins (17% less fluorescence, black dashed line in Figure 4). The quenching, highlighted by the arrow in the figure, suggests that the two proteins come into contact and that the exposed Trp residues are close to the interaction surface, leading to fluorescence quenching. We propose that the quenching stems from the energy transfer from the exposed W155 (or possibly also W168) in FXN to the tryptophan network in SOD2 through the solvent-exposed W181/W186. 

### 3.4. Protein–Protein Docking of FXN and SOD2

To complement the experimental data and gain further insight into the interaction, we decided to model the structure of the FXN:(SOD2)_4_ 1:1 complex using protein–protein molecular docking. We performed both blind docking simulations and simulations using site-specific information from SDSL-EPR and fluorescence as restraints. The structures used for the docking calculations were as follows: FXN (pdb.id 1EKG [15]); SOD2 (pdb.id 5VF9 [49]). 

**Figure 5:**
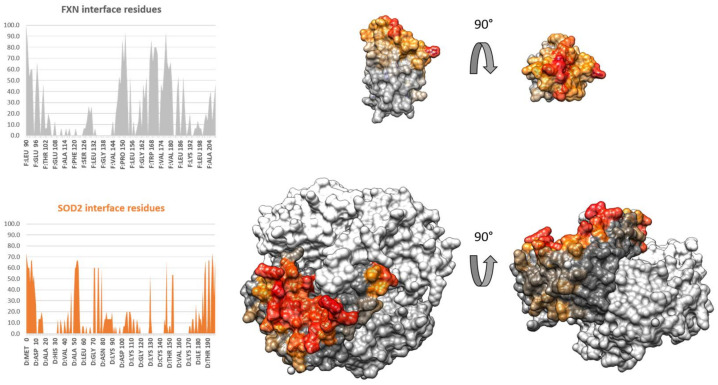
Blind docking simulations FXN/(SOD2)_4_. Left—histogram of the normalized frequency of appearance of each amino acid at the interface: grey, FXN; orange, SOD2. Right—surface mapping of the frequency of appearance at the interface: the frequency with which an individual residue is involved in the interaction lowers going from red to orange; grey residues are never involved in an interaction. SOD2 has only one monomer mapped. FXN is represented with the beta-sheet shown to the reader in the left image.

The top fifteen models obtained from the blind docking simulations were analyzed collectively to identify which residues of FXN and SODs were most often found at the interface of the protein complex. The full details of the docking simulations are reported in the materials section. Briefly, the docking protocol involved three steps: (1) docking simulations using four programs freely available as webservers (ZDOCK [37], ClusPro [36], GRAMM-X [38], PatchDock [39,40]); (2) re-ranking of the solutions using a common scoring function with CONSRank; and (3) analysis of the interface regions in the top 15 re-ranked docking poses using PDBePISA. The normalized frequency with which each residue appeared at the interface in the top 15 models was represented as a histogram and then mapped on the protein surface; the results for the blind docking are shown in Figure 5. In this set of simulations, FXN interacted with a single monomer of SOD2, contacting only a second monomer close to the interface. The SOD2 region involved in the interaction is the hollow region on top of the N and C-terminal tails of the protein on the other side of the active site facing the inner tetrameric cavity. The interaction region for FXN is concentrated mostly around the top part of the protein comprising the N- and C-termini, leaving the beta-sheet region exposed to the solution. These models show a good surface complementarity with several hydrogen bonds and salt bridges being possible; however, they contrast with the experimental evidence. First, a site that is almost always found at the interface is H177 (present in more than 90% of the models), which is in marked contrast to the EPR experimental results, which show limited effects of the complex formation on H177 mobility. Furthermore, the exposed Trp residues for SOD2 are also relatively far away from the interface, as is W155 from FXN, while the other FXN Trp residues are close to the interaction region. Overall, because the Trp residues of the two proteins are far from each other and do not change their solvent exposition, the obtained docking poses make it hard to justify the observed fluorescence quenching.

**Figure 6:**
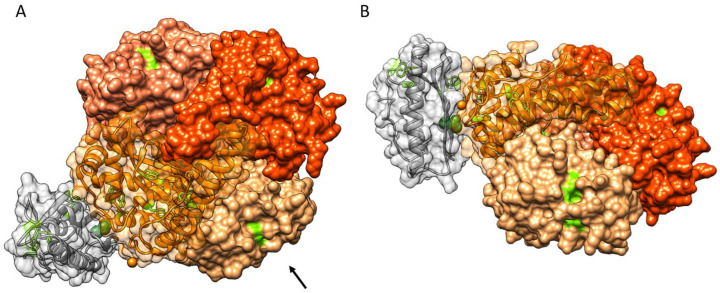
Docking model of the FXN:(SOD2)_4_ 1:1 complex. FXN is shown in grey; SOD2 is shown in shades of orange, one shade per protein chain. Tryptophan residues are shown in light green with stick representation; T133 is shown in dark green with sphere representation. The protein chains involved in the interaction are represented by a partially transparent surface. (**A**) SOD2 viewed in the same orientation as in Figure 4; (**B**) the complex as viewed from the direction of the arrow to better highlight the orientation of FXN.

Given that the blind-docking analysis did not agree with the observations from the spectroscopic data, the docking simulations were also performed using the data as restraints. We chose not to exclude any residue from the interface, even if H177 and S202 could be excluded based on the EPR results. Instead, we opted to locate only W181 and/or W186 from SOD2 close to the binding interface, if possible, as Trp fluorescence was strongly affected. No Trp residue from FXN was chosen, since, while W155 is the only Trp residue that is fully solvent-exposed, W168 is also sufficiently close to the surface and could potentially act as a potential conduit for quenching. In this set of calculations, we specified the Trp residues belonging to a single SOD2 chain as restraints. This is reasonable since the exposed Trp residues of SOD2 are located far from the tetramer interfaces and FXN and, being smaller than a SOD2 monomer, would be unable to reach the other SOD2 monomers when interacting with the Trp region. The best docking model of the restrained simulations is reported in Figure 6 from two different angles. The structure of this complex involves a different region from the blind docking simulations, as expected. The active site of SOD2 is still accessible, as is the iron-binding region of FXN. Although the complementarity of the surface is reduced relative to the blind-docking simulations, and the specific interactions are limited to two hydrogen bonds, this structure satisfies both the fluorescence and the EPR data. FXN now contacts SOD2 using the beta-sheet region, bringing W155 close to the exposed Trp of SOD2; the closest interatom distance between W155 and W186 is only 0.8 nm. Additionally, both H177 and S202 are now far from the region of interaction, which justifies the smaller changes in mobility for these two residues from the EPR data. The full accessibility of the iron-binding region is also in line with experiments conducted in the presence of Fe^3+^.

## 4. Discussion

This study stemmed from previous work by Han et al. [29], which reported on the interaction between yeast FXN and SODs (bovine and human, in that work). To give full meaning to the results, however, the question of whether the same interaction could be detected between human FXN and human SODs needed to be addressed. In this work, we investigated the interaction between the mature form of FXN and mitochondrial SOD2, since Han and coworkers estimated that at physiological concentrations a complex between FXN and SOD1 could not be formed within the mitochondria [29]. Furthermore, since SOD1 is primarily a cytosolic protein, it would probably interact with the immature form of FXN. EPR experiments suggested that human SOD2 interacts with mature FXN. All positions showed a slowdown in dynamics upon the addition of SOD2, which was likely the result of the large increase in the hydrodynamic radius. We did not observe two different spectral populations, suggesting that the proteins undergo fast exchange in solution, and therefore the kinetics of association/dissociation are fast, in the order of tens of nanoseconds. The results of the fluorescence experiments confirm an interaction with SOD2: the fluorescence when both proteins are present was reduced by 17% relative to the sum of the individual contributions. Interestingly, Han et al. observed a ~13% fluorescence enhancement by mixing yeast FXN with human SOD2, as opposed to the quenching we observed. While yeast FXN only has two Trp residues, the diametrically different result suggests that the details of the interaction between human proteins are significantly different from those of the mixed-species system. Together, our experimental results suggest a definite interaction between human FXN and SOD2 in vitro. A quantitative estimation of the K_D_ would require additional experiments and is beyond the scope of the present work. 

Several experimental works point to a possible direct or indirect role of FXN in SOD2 activity, giving a potential physiological relevance for the in vitro interaction that we observed. Both SOD2 and FXN [28] are able to regulate the detoxifying enzymatic mechanisms and inhibit ROS production, and they might act synergistically, since the two proteins are located in the same mitochondrial compartment. Recently, it has been demonstrated that FXN is enriched in the mitochondrial cristae, and its involvement in stabilizing the organization of respiratory chain has been hypothesized based on functional and biochemical analyses [50]. Interestingly, recent cryo-EM studies showed that SOD2 is associated with respiratory supercomplexes in both mycobacteria [51] and *Caenorhabditis elegans* [52], an association that can provide local protection against ROS damage. FXN and SOD2 are both involved in the biochemical hallmarks of FRDA pathophysiology, i.e., increased susceptibility to oxidative stress, iron overload, and a deficit in ISC biogenesis. FXN deficiency correlates with a lower cell antioxidant capacity, especially for SOD2 [53,54]. In the yeast model of FRDA, SOD2 is overexpressed but shows lower activity that can be recovered with manganese supplementation [55]. Furthermore, iron overload plays a role in the inactivation of SOD2, since it can compete with manganese for binding to SOD2, inactivating the enzyme [56,57]. In this context, we propose two hypotheses on the physiological relevance of the FXN/SOD2 interaction, but we cannot exclude the possibility that the link between FXN and SOD2 discussed above is more a consequence of their common involvement in mitochondrial function than the result of a direct interaction. One possibility is that the interaction between the two proteins acts as a modulator of antioxidant mechanism in the vicinity of respiratory complexes, given their localization and their involvement in oxidative stress. A second possibility is that under pathological conditions of excess iron, FXN transiently interacts with SOD2, lowering the local iron concentration, since one FXN can bind several Fe^2+^/Fe^3+^ ions, thus preventing manganese substitution with the consequent inactivation of SOD2. The structure of the proposed complex (Figure 6), in which the iron binding region of FXN is exposed to the solution and does not block the active site of SOD2, could be in line with both hypotheses. 

In conclusion, the results obtained through the combination of SDSL-EPR, fluorescence, and molecular docking prove that mature human FXN interacts with human SOD2 in vitro, confirming the previously observed interaction between yeast FXN and human SOD2. An aspect that should be investigated is whether the currently known pathological variants of FXN affect the interaction. Several involve mutations in the beta-sheet region of FXN (for example, N146K, W155R, R165C), which we found to be important for the interaction with SOD2 as well as with the FeS assembly protein complex. Taking into account the increasing amount of evidence that both proteins are present in the region around respiratory chain complexes and that iron interacts with both, the FXN/SOD2 interaction could be relevant for the protection of FeS clusters during assembly and/or incorporation when FeS clusters are potentially exposed to attacks by reactive oxygen species. All things considered, we suggest that the observed interaction merits further investigation to better frame it within the context of the uncertain physiological role of FXN and the molecular mechanisms of FRDA. 

## Figures and Tables

**Table 1:** Isotropic rotational correlation times for FXN mutants alone (τ_C_^−SOD^) in the presence of 1:1 tetrameric SOD (τ_C_^+SOD^) and their variation (Δτ_C_) obtained from the fitting of the EPR spectra. All values are presented in nanoseconds. The error estimate was derived from the error in the fitting following standard error propagation methods. The g tensor principal components are g_xx_ = 2.0088, g_yy_ = 2.0070, and g_zz_ = 2.0030. The hyperfine tensor principal components are A_xx_ = 0.79 mT, A_yy_ = 0.54 mT, and A_zz_ = 3.68 mT.

Mutant	τ_C_^−SOD^/ns	τ_C_^+SOD^/ns	Δτ_C_ (τ_C_^+SOD^ − τ_C_^−SOD^)/ns
A114C	2.52 ± 0.06	2.96 ± 0.06	0.44 ± 0.09
T133C	2.70 ± 0.06	3.10 ± 0.07	0.40 ± 0.095
H177C	1.13 ± 0.03	1.24 ± 0.03	0.11 ± 0.04
H183C	2.77 ± 0.06	3.33 ± 0.06	0.56 ± 0.10
A193C	2.77 ± 0.06	3.18 ± 0.06	0.41 ± 0.10
S202C	2.30 ± 0.05	2.47 ± 0.05	0.16 ± 0.08

## Data Availability

The data presented in this study are available on request from the corresponding author.

## Supplementary Materials

The following are available online at https://www.mdpi.com/article/10.3390/biomedicines9121763/s1, Figure S1: DS-PAGE followed by Coomassie Brilliant Blue staining for the expression and purification steps of recombinant human FXN90-210, Figure S2: Left - SDS-PAGE followed by Coomassie Brilliant Blue staining. Right - Western Blot analysis, Figure S3: EPR spectra of FXN mutants in the absence (dark red) and in the presence of (SOD2)4 at a 1:0.5 (red) and 1:1 (orange) molar ratio, Figure S4: EPR spectra of FXN mutants in the absence (black) and in the presence (red) of Fe3+, Figure S5: EPR spectra of FXN mutants with Fe3+ in the absence (black) and in the presence (orange) of SOD2, Figure S6: Molecular Dynamics simulation, Table S1: The primer sequences for FXN mutants S202C and H177C.
