# Supplementary material for: A Combined Spectroscopic and In Silico Approach to Evaluate the Interaction of Human Frataxin with Mitochondrial Superoxide Dismutase
Source: Biomedicines. 2021 Nov 25;9(12):1763. doi: 10.3390/biomedicines9121763 (PMC8698469; doi:10.3390/biomedicines9121763)
Supplement: Supplementary file 1 [file biomedicines-09-01763-s001.zip › Bortolus_FXNSOD_ESI.pdf]

Supporting Information

for

A combined spectroscopic and *in silico* approach to evaluate the interaction of human frataxin with mitochondrial superoxide dismutase

Davide Doni[1], Marta Meggiolaro[1,2], Javier Santos[3,4], Gérard Audran[5], Sylvain R. A. Marque[5], Paola Costantini[1], Marco Bortolus[2]*, and Donatella Carbonera[2]

[1] Department of Biology, University of Padova, Viale G. Colombo 3, 35131 Padova, Italy

[2] Department of Chemical Sciences, University of Padova, Via F. Marzolo 1, 35131 Padova, Italy

[3] Instituto de Biociencias, Biotecnología y Biomedicina (iB3-UBA). Departamento de Química Biológica, Facultad de Ciencia Exactas y Naturales, Universidad de Buenos Aires. Intendente Güiraldes 2160 - Ciudad Universitaria, 1428EGA CONICET, Godoy Cruz 2290, C1425FQB. C.A.B.A. Argentina

[4] Instituto de Química y Fisicoquímica Biológicas, Dr. Alejandro Paladini, Universidad de Buenos Aires, CONICET, Junín 956, 1113AAD C.A.B.A., Argentina

[5] Aix Marseille Universitè, CNRS, ICR, UMR 7273, case 551, Ave Escadrille Normandie Niemen, 13397 Marseille Cedex 20, France

*to whom correspondence should be addressed: marco.bortolus@unipd.it

1) Mutagenesis primers for FXN H177C and S202C

In Table S1 below, we report the primer sequences for FXN mutants S202C and H177C used in this study for SDSL.

Table S1: The primer sequences for FXN mutants S202C and H177C.

FXN mutant	Primer name	Primer sequence
FXN_H177C	FXN_H177C_for	5' - GGTGTACTCCTGCGACGGCGTGT - 3'
	FXN_H177C_rev	5' - CAGTTTTTCCCAGTCCAGTC - 3'
FXN_S202C	FXN_S202C_for	5' - GGACTTGTCTTGCTTGGCCTATT - 3'
	FXN_S202C_rev	5' - AGTTTGGTTTTTAAGGCTTTAGTG - 3'

2) FXN expression and purification

In the Figure S1 below, we report a SDS-PAGE gel showing the different steps of expression and purification of recombinant human $FXN_{90\text{-}210}$. FXN mutants for SDSL have been expressed and purified with the same procedure.

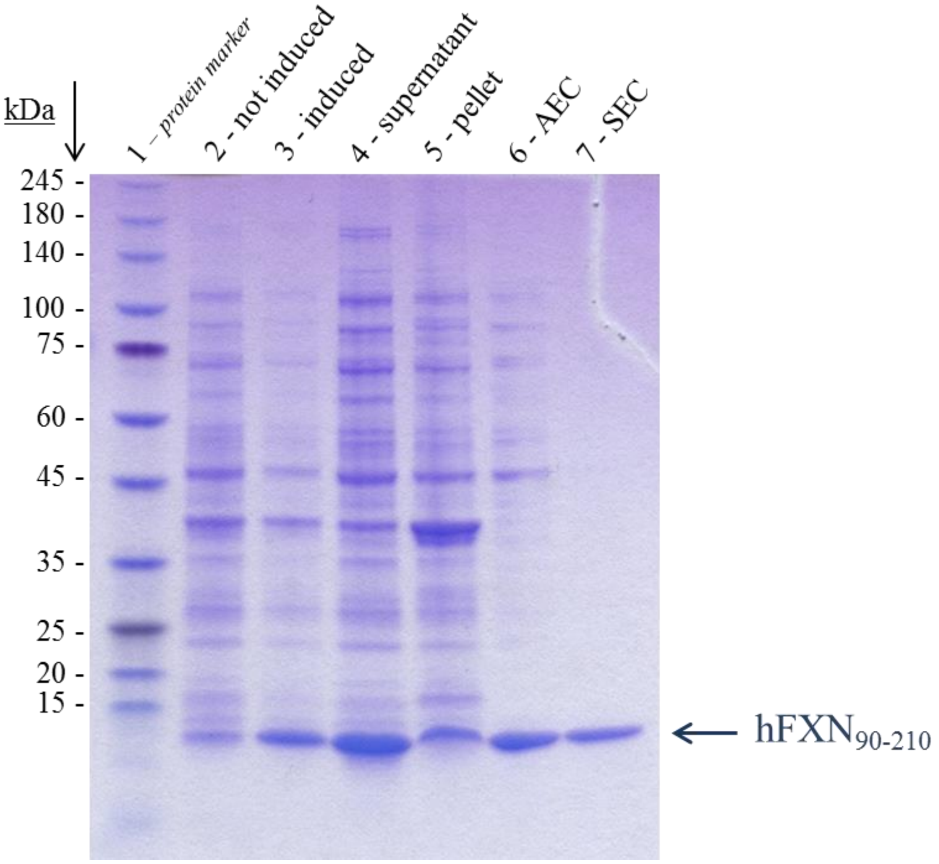

Figure S1. SDS-PAGE followed by Coomassie Brilliant Blue staining for the expression and purification steps of recombinant human $FXN_{90\text{-}210}$. Lane (1): molecular weight protein ladder. Lanes (2) and (3) correspond to the bacterial culture before and after the induction with IPTG, respectively. Lane (4) and (5) represent the soluble and insoluble fraction of the total cellular lysate. Lane (6) corresponds to the protein pool after the first purification step performed by anionic exchange chromatography (AEC). Lane (7) corresponds to the protein pool after the second purification step performed by size exclusion chromatography (SEC).

3) SOD2 purity and identity control

In Figure S2 below, we report the SDS-PAGE gel and relative Western Blot analysis of recombinant human SOD2 performed to verify the purity and identity of SOD2 used in EPR and fluorescence spectroscopy experiments. The SDS-PAGE gel shows that recombinant SOD2 is present in high purity, and the Western Blot confirmed the identity of the protein.

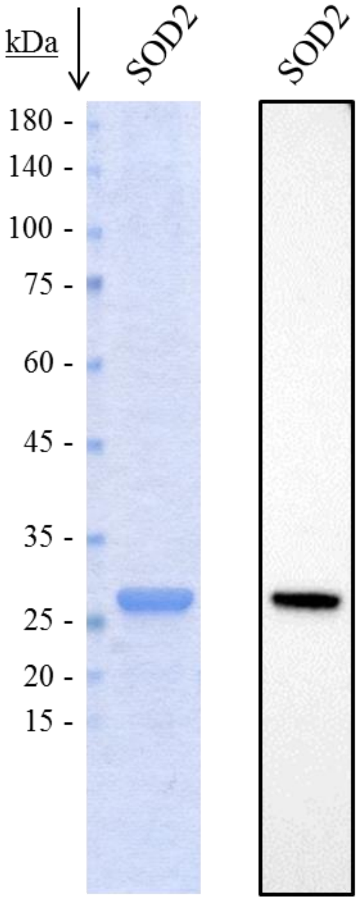

Figure S2. Left - SDS-PAGE followed by Coomassie Brilliant Blue staining. Right - Western Blot analysis.

4) FXN:SOD2 experiments at different ratios

The effects of two different ratio of FXN:SOD2 on the mobility of the spin labels of FXN mutants are reported in Figure S3. FXN and SOD2 have been mixed at a 1:0 (dark red), 1:0.5 (red), and 1:1 (orange) ratio. The spectra show a progressive slowdown at all spin label positions, but the spectral shape does not significantly change, a sign that most of the proteins are already involved in the complex.

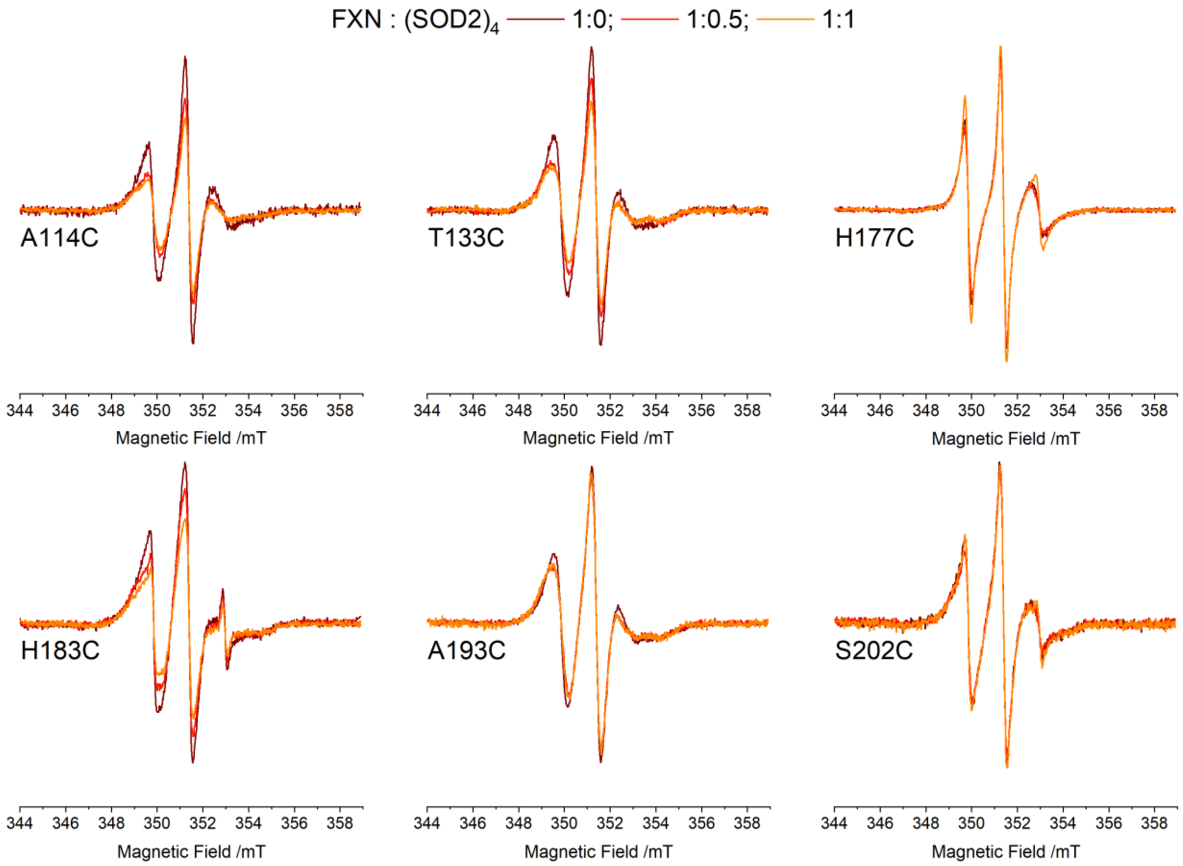

Figure S3. EPR spectra of FXN mutants in the absence (dark red) and in the presence of $(SOD2)_4$ at a 1:0.5 (red) and 1:1 (orange) molar ratio. All spectra have been normalized to the same number of spins to compare the spectral shape in terms of spin probe mobility.

5) FXN/SOD2 experiments with Fe^{3+}

The effects of Fe^{3+} on FXN in the presence/absence of SOD2 have been evaluated using EPR spectroscopy. Previously, we showed that the addition of excess iron slows down FXN motion by inducing its aggregation at all positions [noi].

Here, we show in Figure S4 that the effects are still present also for the new mutated positions (H177 and S202) with the formation of new slow components on the side wings of the spectra (red spectra).

The effects of Fe^{3+} in the presence of SOD2 are reported in Figure S5. FXN and SOD2 have been mixed, and then Fe^{3+} has been added. The spectra do not change significantly when SOD2 is present (orange spectra).

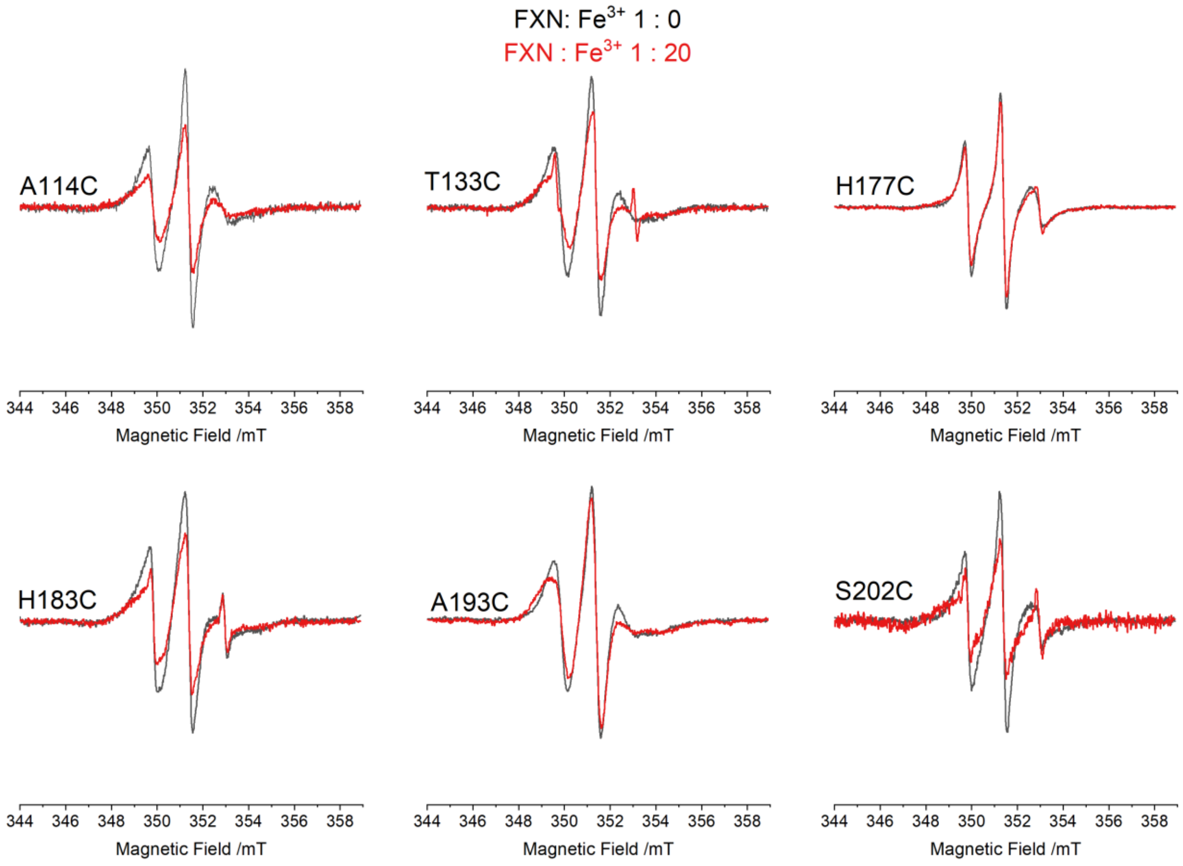

Figure S4. EPR spectra of FXN mutants in the absence (black) and in the presence (red) of Fe^{3+}. [FXN] = 10 μM; [Fe^{3+}] = 0/200 μM. All spectra have been normalized to the same number of spins to compare the spectral shape in terms of spin probe mobility.

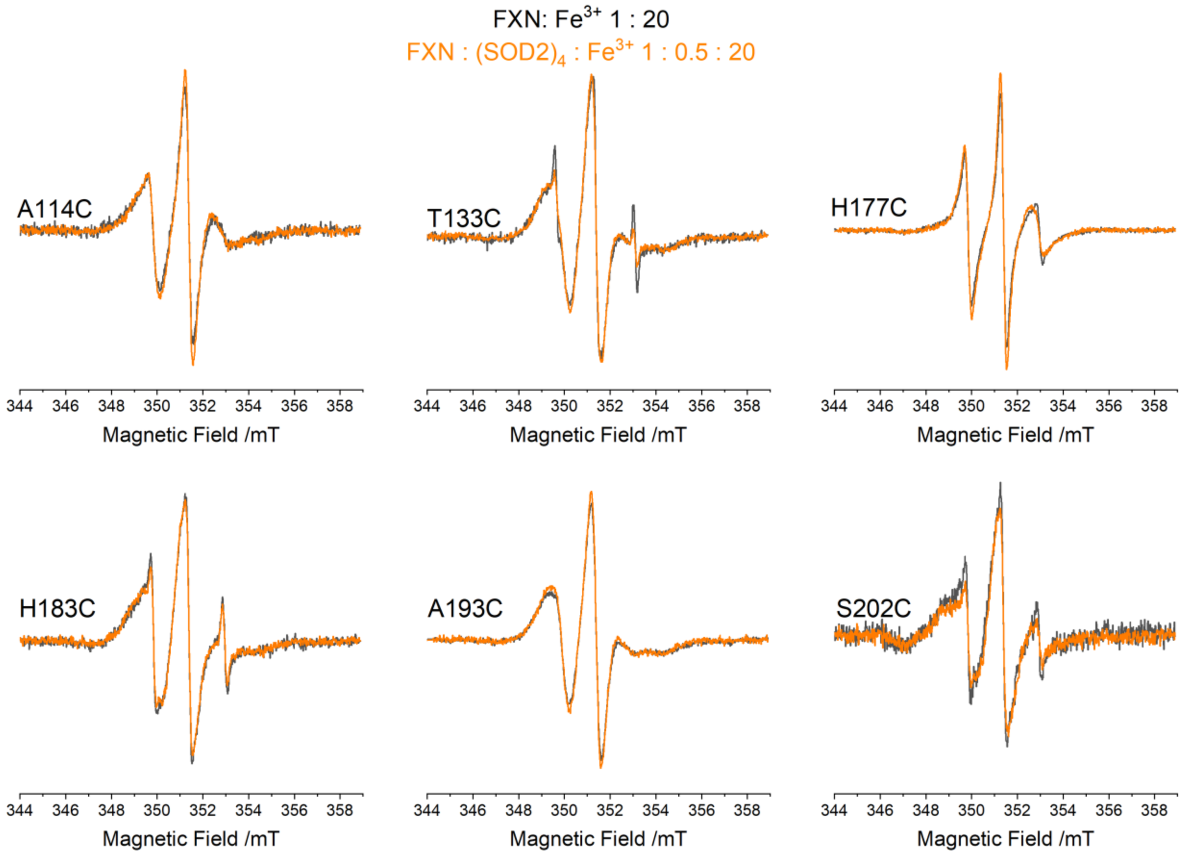

Figure S5. EPR spectra of FXN mutants with Fe^{3+} in the absence (black) and in the presence (orange) of SOD2. [FXN] = 10 µM; [SOD2] = 0/20 µM; [Fe^{3+}] = 200 µM. Black, FXN:$(SOD2)_4$:Fe^{3+} 1:0:20; orange, FXN:$(SOD2)_4$:Fe^{3+} 1:0.5:20. All spectra have been normalized to the same number of spins to compare the spectral shape in terms of spin probe mobility.

6) Molecular Dynamics simulation

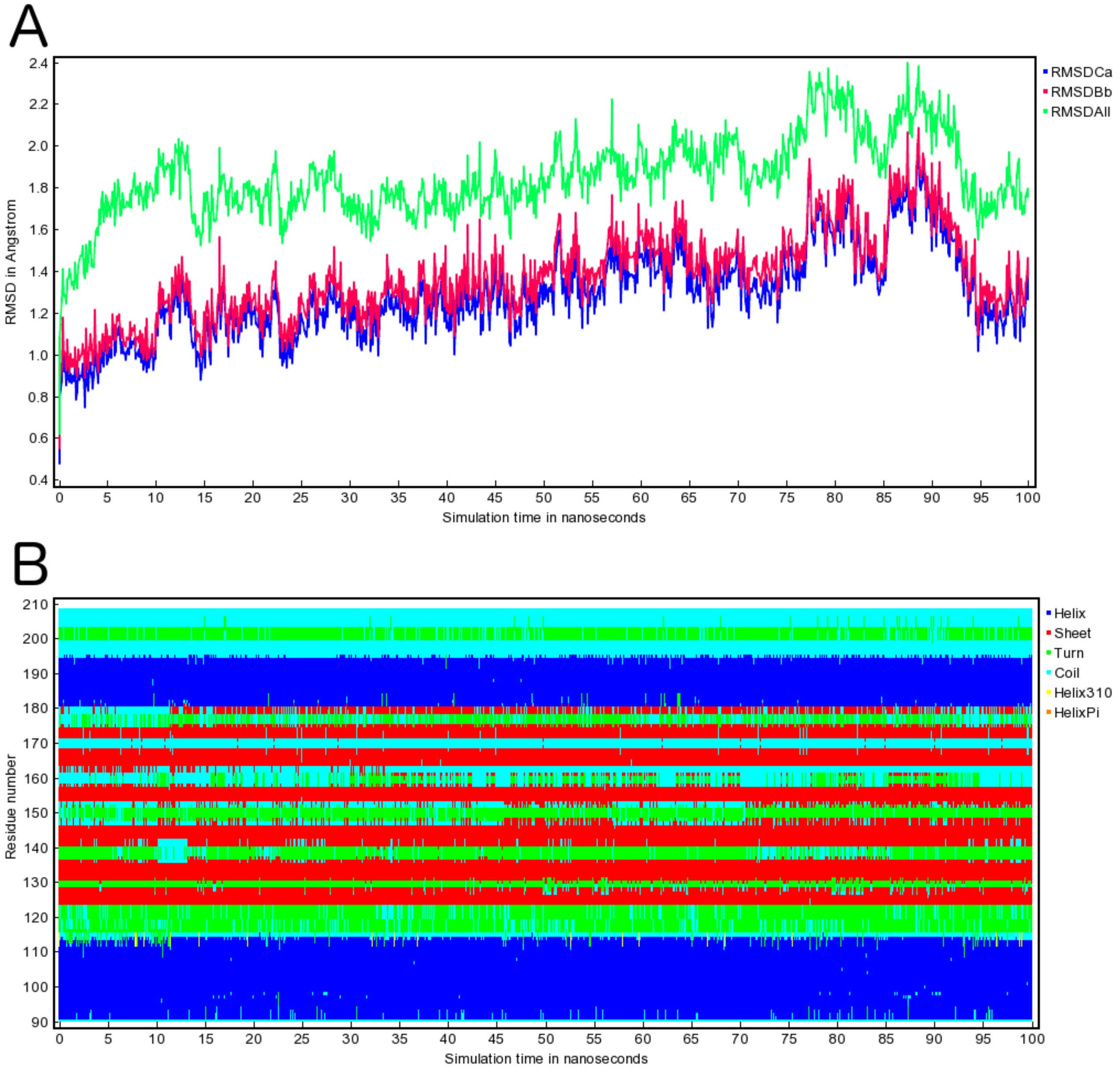

Figure S6. Molecular Dynamics simulation. (A) The root-mean-square deviation (RMSD). The plot shows Calpha (RMSDCa), backbone (RMSDBb) and all-heavy atom (RMSDAll]). (B) The secondary structure content (B) along the simulation time. The analysis of production runs was carried out using YASARA Structure [1].

[1] H. Land, M.S. Humble, YASARA: A Tool to Obtain Structural Guidance in Biocatalytic Investigations, Methods Mol Biol 1685 (2018) 43-67.
